# Cerium enhances germination and shoot growth, and alters mineral nutrient concentration in rice

**DOI:** 10.1371/journal.pone.0194691

**Published:** 2018-03-26

**Authors:** Sara Monzerrat Ramírez-Olvera, Libia Iris Trejo-Téllez, Soledad García-Morales, Juan Antonio Pérez-Sato, Fernando Carlos Gómez-Merino

**Affiliations:** 1 Department of Biotechnology, Colegio de Postgraduados Campus Córdoba, Amatlán de los Reyes, Veracruz, Mexico; 2 Department of Soil Science, Laboratory of Plant Nutrition, Colegio de Postgraduados Campus Montecillo, Texcoco, State of Mexico, Mexico; 3 Department of Plant Biotechnology, CONACYT-Center for Research and Assistance in Technology and Design of the State of Jalisco (Centro de Investigación y Asistencia en Tecnología y Diseño del Estado de Jalisco), Zapopan, Jalisco, Mexico; Hainan University, CHINA

## Abstract

Cerium (Ce) belongs to the rare earth elements (REEs), and although it is not essential for plants, it can stimulate growth and other physiological processes. The objective of this research was to evaluate the effect of Ce on seed germination, initial seedling growth, and vegetative growth in rice (*Oryza sativa* L.) cv. Morelos A-98. During the germination process, the seeds were treated with Ce concentrations of 0, 4, 8, and 12 μM; after 5 d, germination percentage was recorded and after 10 d seedling growth was measured. For vegetative growth, a hydroponic system was established where 14-d-old plants without previous Ce treatment were transferred into nutrient solution. After two weeks of acclimatizing, 0, 25, 50, and 100 μM Ce were added to the nutrient solution for 28 d. Ce significantly increased germination and the initial growth variables of the seedlings. During vegetative growth, Ce increased plant height, number of tillers, root volume, and shoot fresh and dry biomass, without affecting root biomass weight. With low Ce concentrations (25 and 50 μM), the concentrations of chlorophylls and amino acids in the shoots were similar to those in the control, like amino acid concentration in the roots at a concentration of 25 μM Ce. Conversely, the concentration of total sugars increased in the shoot with the application of 25, 50, and 100 μM Ce, and in the roots with the application of 50 μM Ce. Also, Ce did not affect the concentration of macro or micronutrients in the shoots. However, in the roots, the high Ce concentration decreased the concentrations of Ca, Fe, Mn, and Zn, while the Mg concentration increased. Our results indicate that Ce, at the right concentrations, can function as a biostimulant in rice germination and growth.

## Introduction

The rare earth elements (REEs) consist of 17 elements: lanthanum (La), cerium (Ce), praseodymium (Pr), neodymium (Nd), promethium (Pm), samarium (Sm), europium (Eu), gadolinium (Gd), terbium (Tb), dysprosium (Dy), holmium (Ho), erbium (Er), thulium (Tm), ytterbium (Yb), lutetium (Sc), and yttrium (Y), all of them showing similar physicochemical characteristics [1]. Of these elements, Ce represents 0.0043% of the Earth’s crust, making it the most abundant REE on the planet [2, 3]. In soils, it is found in similar concentrations to those of Cu and Zn [4, 5], 20–60 mg kg^-1^. Nevertheless, these concentrations can vary depending on the mineral characteristics of the bedrock and on human impact; in anthropic environments, it is found in higher concentrations [6].

Like other REE, Ce has been used in agriculture where it has shown diverse effects. Among the positive effects are significant increases in germination [7], plant height [8], root growth and volume [9–11], biomass weight [7, 8, 12, 13, 14], yield [15], chlorophyll concentration [16, 17], total sugars [15, 18], and the nutrimental status of several species [9, 5, 17, 19]. Nevertheless, Ce can also reduce root length and dry matter weight both of the shoots and roots, as well as the nutrient content in wheat (*Triticum aestivum*) [20]. At a low soil pH (i.e. 4.08), Ce decreases the germination of radish (*Raphanus sativus*) and tomato (*Solanum lycopersicum*) seeds, which is associated with higher mobility and availability of the element in the soil [21]. Ce can also interfere in the absorption of K, Mg, Ca, Na, Fe, Mn, Zn, Cu, and Mo in roots and shoots [9, 5]. Moreover, the application of Ce to the soil can promote the assimilation of N and increase the activity of the photosystem II (PSII), thus stimulating plant development [22]. The effect of Ce on plant metabolism and physiology depends on the applied dose, time of exposition, management conditions, species, and vegetative stage. In cereals like rice [23], wheat [24], and barley (*Hordeum vulgare*) [25], the application of cerium oxide nanoparticles (*n*CeO_2_) has been studied. In rice, the application of 500 mg *n*CeO_2_·kg^-1^ to the soil decreased the amount of starch in the grain in two of the three species evaluated, while K and Ca concentrations in rice increased and S and Fe concentrations decreased [23].

Rice is the second most produced cereal in the world after maize (*Zea mays*), and its consumption has increased in the last few years [26]. According to projections reported by Moyer [27], the world will face harsher effects of global climate change in the next 50 years compared with current-day estimates, resulting in decreased agricultural production in some countries. By the year 2050, the world population is expected to reach 9 billion, which will put great pressure on natural resources and possibly worsen environmental deterioration, thus threatening food security and agricultural sustainability worldwide. Hence, the study of beneficial elements like Ce as an inductor of plant adaptive responses and a biostimulant improving crop production and productivity under restrictive conditions imposed by climate change is of paramount importance. The objective of this research was to evaluate the effect of Ce on the germination, initial seedling growth, and vegetative growth of rice cv. Morelos A-98, as well as its effect on the concentrations of chlorophyll, total amino acids, soluble sugars, and macro and micronutrients.

## Materials and methods

### Plant material and seed disinfection

The rice cultivar Morelos A-98 (*Oryza sativa* L. ssp. *indica*) was used. It was obtained from the Germplasm Bank of the Mexico’s National Institute of Forestry, Agriculture, and Livestock Research (Instituto Nacional de Investigaciones Forestales, Agrícolas y Pecuarias—INIFAP), located in the Zacatepec Experimental Station, Morelos, Mexico (18°39’ NL, 99°12’ WL, 910 masl).

The rice seeds were submerged in 70% ethanol for 10 min and rinsed three times with sterile distilled water. Subsequently, they were immediately left to soak for 1 h in 5% NaClO, with one drop of Tween^®^ 20 (Hycel, Zapopan, Mexico). Afterwards, the seeds were rinsed five times with sterile distilled water. Finally, the seeds were dried on filter paper.

### Initial germination and growth

Sterile filter paper was placed in lidded plastic containers (12 x 11 cm), superficially disinfected with 70% ethanol. The treatments consisted of applying 15 mL of either 0, 4, 8, or 12 μM Ce from CeCl_3_ 7H_2_O (Sigma-Aldrich; Saint Louis, MO, USA), plus the control which was sterile distilled water. To avoid any possible fungal contamination, 0.2% (w/v) methyl 2-benzimidazole carbamate was added to all treatments, including the control. Subsequently, 25 disinfected seeds were placed in each container, leaving enough space between them to allow root elongation. The containers were closed and placed in the dark at 28 °C for 3 d, after which they were exposed to natural light. There were three replicates (3 containers) per treatment.

Germination records were made daily for 5 d, considering as germinated the seeds with a radicle over 2 mm long. Ten days after sowing, seedling height, root length, number of roots, and shoot and root fresh and dry biomass weights were recorded.

### Vegetative growth

Previously disinfected rice seeds were placed in glass flasks with MS medium (Sigma-Aldrich; Steinheim, Germany) supplemented with 3% (w/v), sucrose (J. T. Baker; Philadelphia, PA, USA), and solidified with 0.8% agar (Merck; Darmstadt, Germany). The flasks were placed in the dark at 28 °C for 3 d, and then exposed to natural light (12 hours photoperiod at 520 μmol m^-2^ s^-1^ light intensity) for 11 d.

Subsequently, the 12-d-old rice seedlings were transferred to a hydroponic system in 14 L containers with Magnavaca nutrient solution modified by Famoso *et al*. [28] with the following concentrations: 1 mM KCl, 1.5 mM NH_4_NO_3_, 1 mM CaCl_2_ 2H_2_O, 45 μM KH_2_PO_4_, 200 μM MgSO_4_ 7H_2_O, 500 μM Mg(NO_3_)_2_ 6H_2_O, 155 μM MgCl_2_ 6H_2_O, 11.8 μM MnCl_2_ 4H_2_O, 33 μM H_3_BO_3_, 3 μM ZnSO_4_ 7H_2_O, 0.8 μM CuSO_4_ 5H_2_O, 1 μM NaMoO_4_ 2H_2_O, and 77 μM Fe-EDTA. Seven days after transplant, the Magnavaca solution was replaced with Yoshida solution (1.43 mM NH_4_NO_3_, 1.00 mM CaCl_2_ 2H_2_O, 1.64 mM MgSO_4_ 7H_2_O, 1.32 mM K_2_SO_4_, 320 μM NaH_2_PO_4_, 100 μM Fe-EDTA, 7.99 μM MnCl_2_ 4H_2_O, 0.15 μM ZnSO_4_ 7H_2_O, 0.15 μM CuSO_4_ 5H_2_O, 0.08 μM (NH_4_)_6_Mo_7_O_24_ 4H_2_O, and 1.39 μM H_3_BO_3_) [29]. Fourteen days after transplant, 0, 25, 50, or 100 μM Ce from CeCl_3_ 7H_2_O were applied with the nutrient solution. The nutrient solution was completely replaced every 7 d, while water consumed by the plant was replaced every three days. The solution pH was adjusted to 5.5 using H_2_SO_4_ or NaOH 1 N. The experiment was carried out in a greenhouse with a mean temperature of 28 °C/16 °C (day/night), relative humidity of 57%, and 16 h light (745 μmol m^-2^ s^-1^) and 8 h darkness.

At 28 d after the beginning of the treatments, the plants were removed from the nutrient solution, rinsed, and the following variables were recorded: plant height, root volume and length, number of tillers, and fresh biomass weight. The plants were separated into shoot and roots, and subsequently dried in a forced-air stove (Riossa HCF-125; Monterrey, Mexico) at 72 °C for 72 h. Then the dry matter weight was determined.

### Nutrimental analysis

Nutrimental analysis was performed following the protocols described by Alcántar and Sandoval (1999) [30]. First, 0.25 g dry tissue from shoots and roots were taken and subjected to wet digestion with a mixture of H_2_SO_4_:HClO_4_ (2:1, v:v). After digestion, the sample was taken to 25 mL volume with de-ionized water and filtered. The N concentration was determined through the micro-Kjeldahl method, and the rest of the elements through readings of the extracts from the digestion in an inductively coupled plasma optical emission spectrophotometer (Varian ICP OES 725-ES; Mulgrave, Australia).

### Concentrations of chlorophylls and total free amino acids

Chlorophylls concentrations were determined according to Geiger et al. (1998) [31]. The shoots and roots were ground separately using liquid nitrogen. From each tissue, 60 mg fresh tissue were weighed, and a triple ethanol extraction was done (80, 80, and 50%). In each extraction, the samples were incubated in a water bath at 80 °C for 20 min and centrifuged at 14000 rpm for 5 min. The supernatants of each extraction were recovered and mixed. Concentrations of chlorophyll *a* and *b* were determined in the shoot by reading the extracts at 635 and 645 nm in a spectrophotometer (Jenway 6715; Staffordshire, UK). Concentrations of total free amino acids were determined in the shoots and roots using the ninhydrin method [32], and the samples were analyzed at 570 nm. L-leucine (Sigma-Aldrich; Steinheim, Germany) was used to estimate the standard curve.

### Concentrations of total soluble sugars

Quantification of total soluble sugars in plant tissues was determined following the protocol described by Bailey (1958) [33]. Accordingly, from the shoots and roots, 500 mg were weighted, and extraction was done using 50 mL 80% ethanol on a hotplate stirrer. The supernatant was filtered and taken to a final volume of 20 mL. Subsequently, 1 mL of the obtained extract was taken and 5 mL 0.4% (w/v) anthrone in concentrated H_2_SO_4_ (Merck; Darmstadt, Germany) were added. During this whole process, the samples were placed on ice. After this, the samples were incubated in a water bath at 95 °C for 15 min; the reaction was ended by placing the samples in ice. To quantify total sugars, a standard curve was done using glucose (Sigma-Aldrich; Saint Louis, MO, USA), and measured at an absorbance of 600 nm.

### Statistical analysis

An analysis of variance was performed, and the means were compared with the Duncan test, with a 0.05 degree of significance. Also, a Pearson correlation between the evaluated variables and the Ce concentration was done in shoots and roots, with a 0.05 degree of significance. The SAS 9.3 [34] software was used for both tests.

## Results

### Ce increases seed germination, and stimulates growth and biomass production of rice in its initial development stage

**Fig 1:**
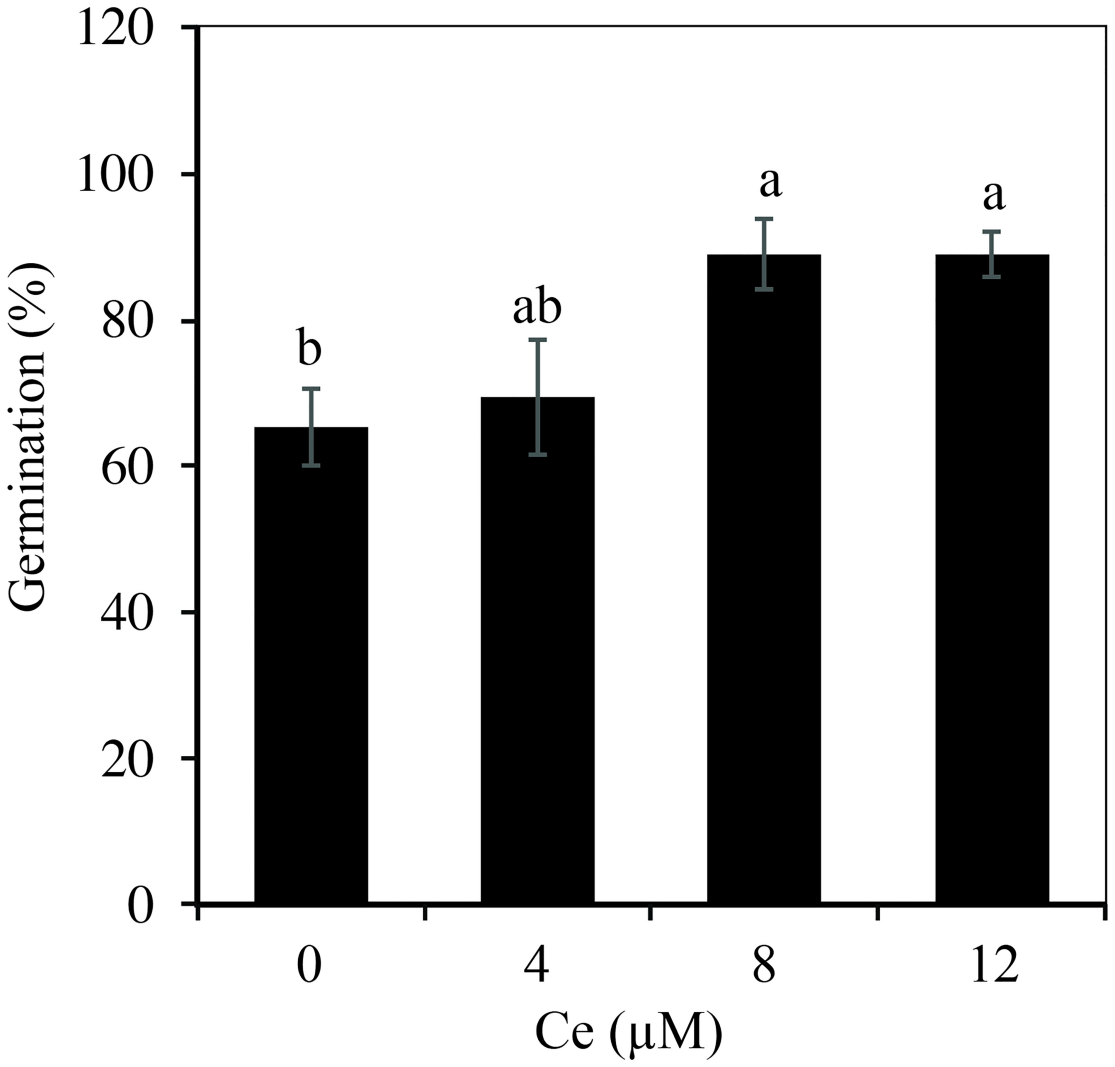
Germination percentage of rice seeds exposed to different Ce concentrations. Rice seeds were treated with 0, 4, 8, or 12 μM Ce. Cerium was supplied as CeCl_3_ 7H_2_O. Data presented correspond to day 5 after establishing the experiment. The figure represents the mean of three independent replicates, each replicate with 25 seeds. Means ± SD with different letters indicate statistical differences among treatments (Duncan *P* ≤ 0.05).

The germination percentage of rice cv. Morelos A-98 seeds increased as the Ce concentration in the solution was raised (Fig 1). When applying 8 and 12 μM Ce, the germination percentage increased by 36.2% with respect to the control, while with 4 μM Ce, there were no significant differences with respect to the control.

**Fig 2:**
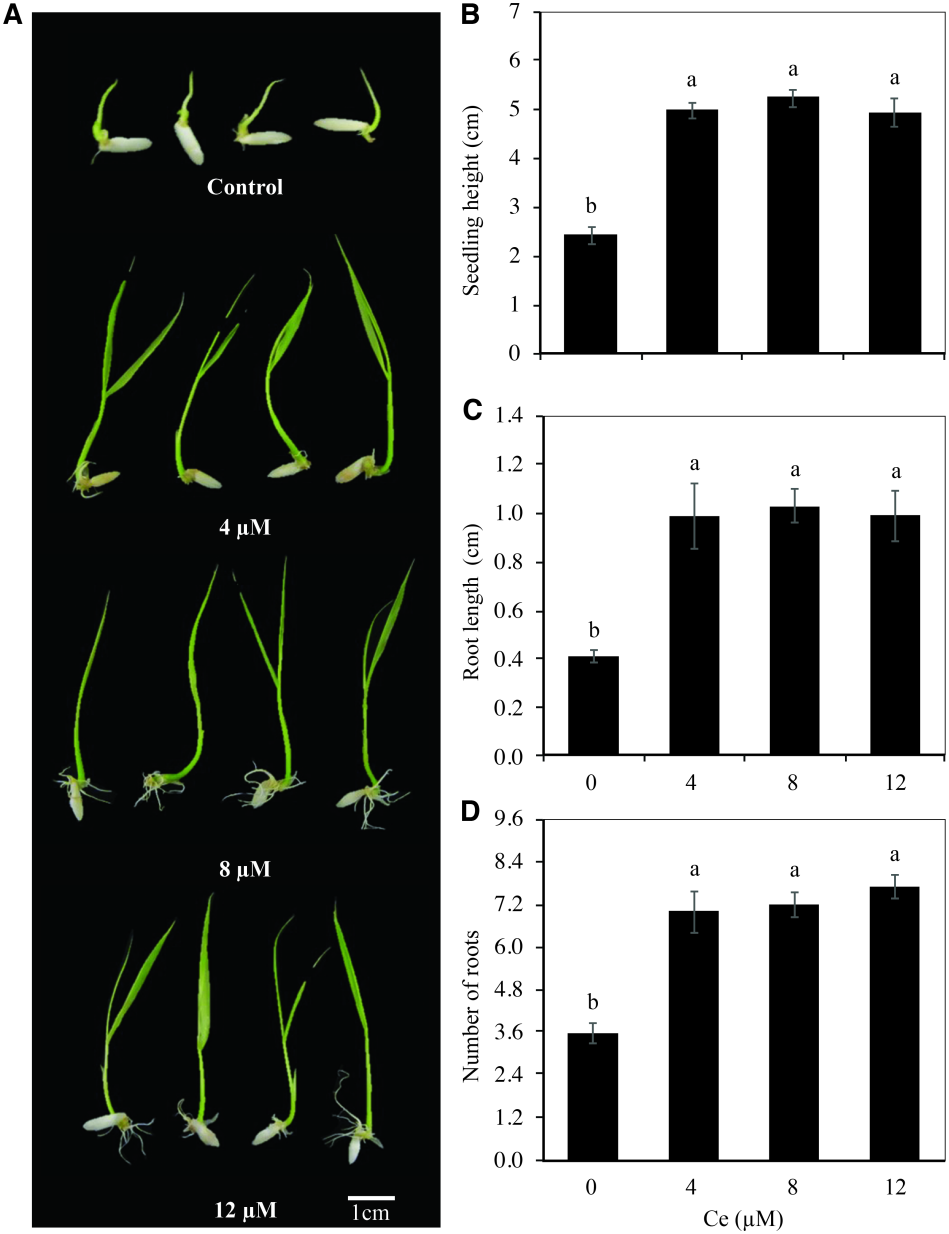
Growth of rice seedlings exposed to different Ce concentrations. Rice seedlings were exposed to 0, 4, 8, or 12 μM Ce. Cerium was supplied as CeCl_3_ 7H_2_O. Photographs show seedling growth (A), plant height (B), root length (C), and number of roots (D) of rice cv. Morelos A-98 seedlings. The growth variables were determined 10 days after the establishment of the experiment. The figure represents the mean of three independent replicates; eight seedlings were measured in each replicate. Means ± SD with different letters indicate statistical differences among treatments (Duncan, *P* ≤ 0.05).

Ce application visibly stimulated rice seedling growth (Fig 2A). Seedling height (Fig 2B) and root length (Fig 2C) increased significantly by over 100% with the addition of 4, 8, and 12 μM Ce, compared to the control. Moreover, the number of roots doubled with the application of 12 μM Ce, and 95% more roots were counted with concentrations of 4 and 8 μM Ce (Fig 2D), relative to the control. Seedling height, root length, and number of roots showed no statistical differences between the three Ce concentrations tested but were statistically superior to the control.

**Fig 3:**
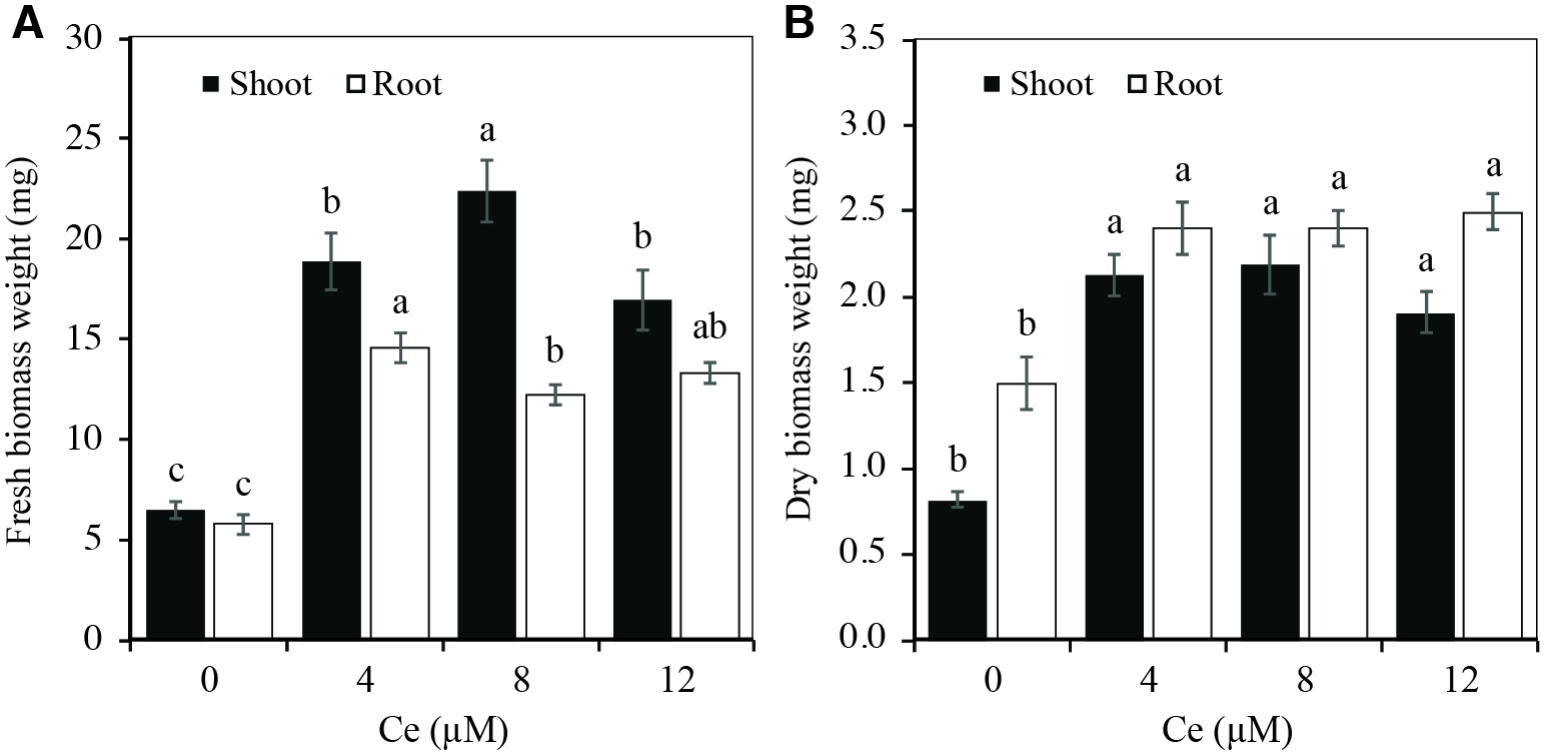
Biomass production of rice seedlings exposed to different Ce concentrations. Fresh (A) and Dry (B) Biomass Weights of shoots and roots of rice cv. Morelos A-98 seedlings treated with 0, 4, 8, or 12 μM Ce. Cerium was supplied as CeCl_3_ 7H_2_O. Biomass production was determined 10 days after establishing the experiment. The figure represents the mean of three independent replicates; eight seedlings were measured in each replicate. Means ± SD with different letters in each variable indicate statistical differences among treatments (Duncan, *P* ≤ 0.05).

The fresh biomass weight of shoots and roots of rice seedlings increased significantly with the application of Ce. This increase was greater in the treatment consisting of 8 μM Ce in shoots and 4 μM Ce in roots (Fig 3A). Similar results were obtained in dry biomass of both shoots and roots (Fig 3B).

### Ce increases the number of tillers and biomass production during the vegetative growth of rice

Once it was observed that Ce increased the germination percentage and rice growth during the seedling stage, the effect of this element on the vegetative growth of rice plants was assessed. To do this, 26-d-old plants were exposed to increasing doses of Ce (0, 25, 50, and 100 μM), which were added to the nutrient solution. Cerium was supplied as CeCl_3_ 7H_2_O too.

**Fig 4:**
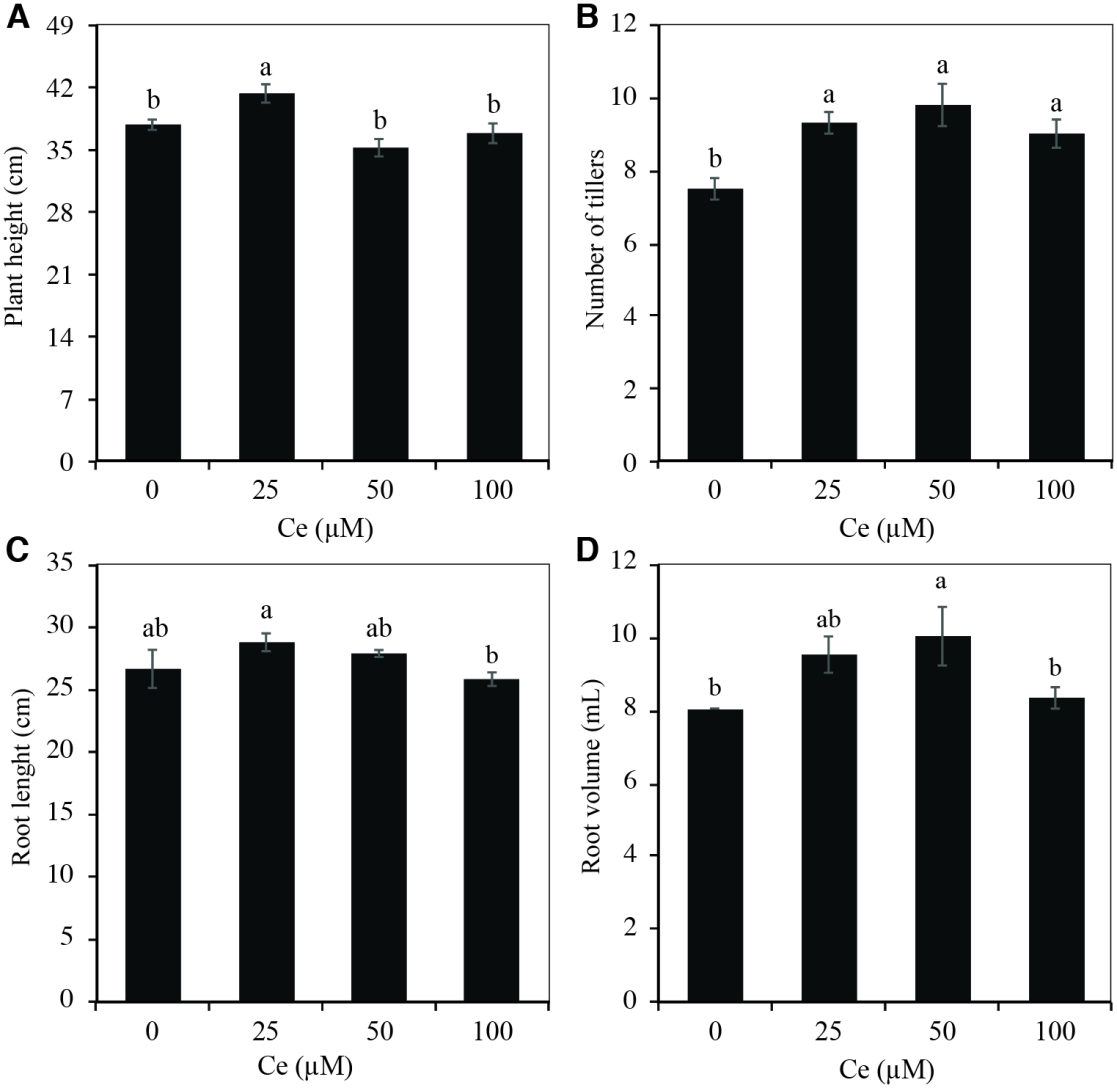
Growth of rice plants exposed to different Ce concentrations. Plant height (A), number of tillers (B), root length (C), and root volume (D) of rice cv. Morelos A-98 plants treated with 0, 4, 8, or 12 μM Ce. Cerium was supplied as CeCl_3_ 7H_2_O. Bars represent means of 26-d-old plants grown in Yoshida nutrient solution for 28 d. Means ± SD with different letters in each variable indicate statistical differences among treatments (Duncan, *P* ≤ 0.05).

Plant height was statistically greater than that of the control only when plants were treated with 25 μM Ce (Fig 4A), while treatments with 50 and 100 μM Ce reduced this variable. Interestingly, the number of tillers increased by 23.3, 30, and 20% with the application of 25, 50, and 100 μM Ce, respectively, compared to the control (Fig 4B). Conversely, root length was not influenced by the Ce concentrations evaluated (Fig 4C). When applying 25 μM Ce, root volume increased by 20% with respect to the control, although the difference was not significant; however, when 50 μM Ce were applied, the root volume increased by 25% as compared to the control, and this difference was significant (Fig 4D).

**Fig 5:**
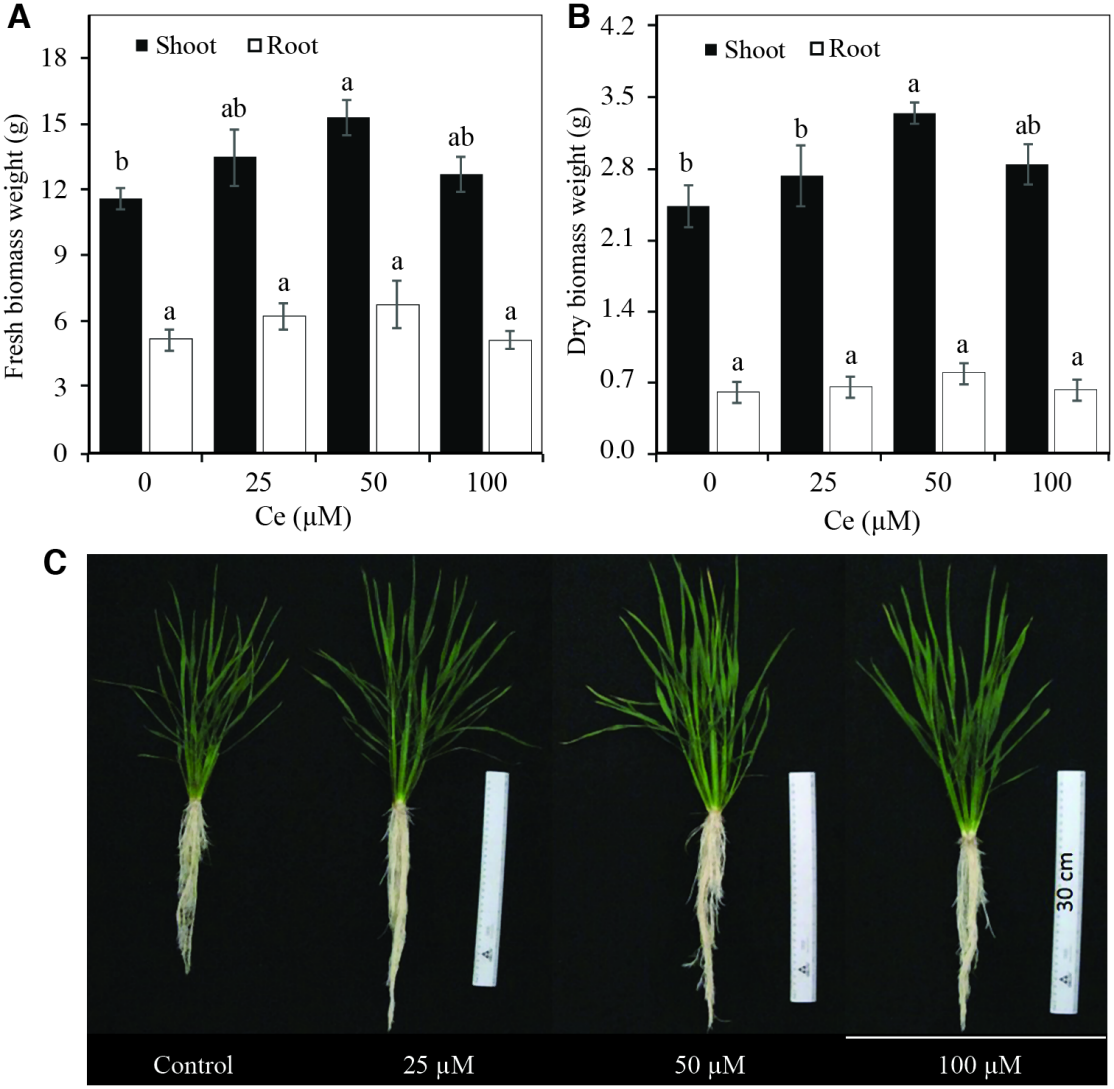
Fresh and dry biomass weight and plant growth of rice plants in response to the application of different Ce concentrations. Fresh (A) and Dry (B) Biomass Weights of shoots and roots, and growth (C) of rice cv. Morelos A-98 plants treated with 0, 25, 50, or 100 μM Ce. Cerium was supplied as CeCl_3_ 7H_2_O. Bars represent means of 26-d-old plants grown in the nutrient solution for 28 d. Means ± SD with different letters in each variable indicate statistical differences among treatments (Duncan, *P* ≤ 0.05).

The shoot fresh biomass weight (SFBW) increased by 16.3, 31.8, and 9.5% with the application of 25, 50, and 100 μM Ce, respectively, in comparison with the control. However, only with 50 μM were there significant differences with respect to the control (Fig 5A). Likewise, the shoot dry biomass weight (SDBW) increased by 12.3, 37.4, and 12.9% in the treatments with 25, 50, and 100 μM Ce, but only the results with the 50 μM Ce concentration were statistically different from the control (Fig 5B). Contrary to the effect on shoots, this element had no influence on root fresh biomass weight (RFBW) or root dry biomass weight (RDBW) (Fig 5A and 5B).

In general, in the 0 to 50 μM Ce concentration interval, there were positive responses in the accumulation of fresh and dry matter in the shoots, the number of tillers, and in root volume.

### Cerium decreases chlorophylls and amino acid concentrations and increases sugars concentrations

**Fig 6:**
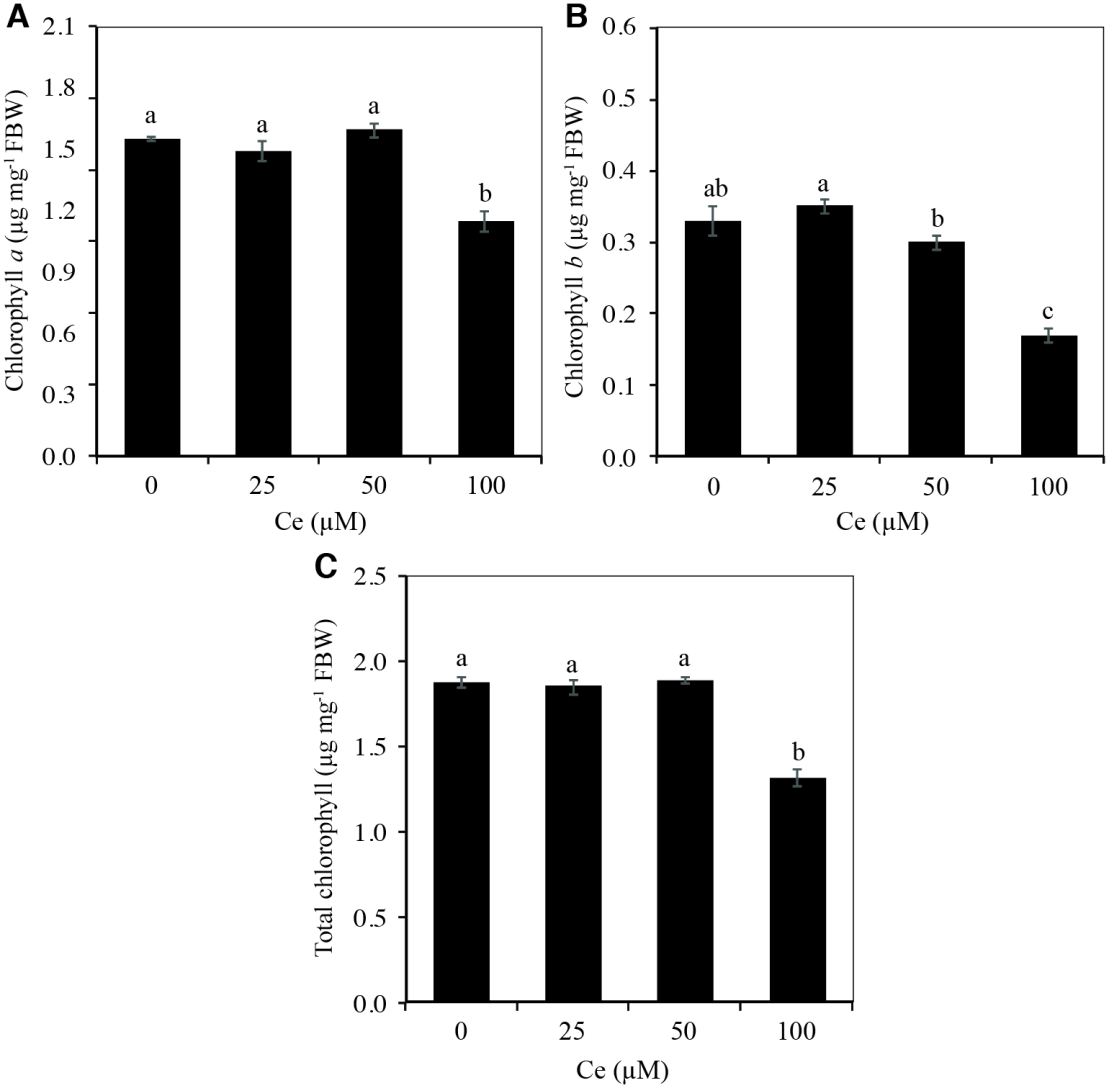
Chlorophyll concentrations in rice leaves in response to the application of Ce. Concentration of chlorophyll *a* (A), chlorophyll *b* (B), and total chlorophylls (C) in shoots of rice cv. Morelos A-98 plants treated with 0, 25, 50, or 100 μM Ce. Cerium was supplied as CeCl_3_ 7H_2_O. Bars represent means of 26-d-old plants grown in Yoshida nutrient solution for 28 d. Means ± SD with different letters in each variable indicate statistical differences among treatments (Duncan, *P* ≤ 0.05).

The concentration of chlorophyll *a* in shoots decreased significantly with the application of 100 μM Ce, in comparison with the control (Fig 6A). Likewise, this treatment decreased the concentration of chlorophyll *b* by 49.2% with respect to the control (Fig 6B). With regard to the total chlorophyll concentration, it decreased by 30.1% with the application of 100 μM Ce in comparison with the control (Fig 6C).

**Fig 7:**
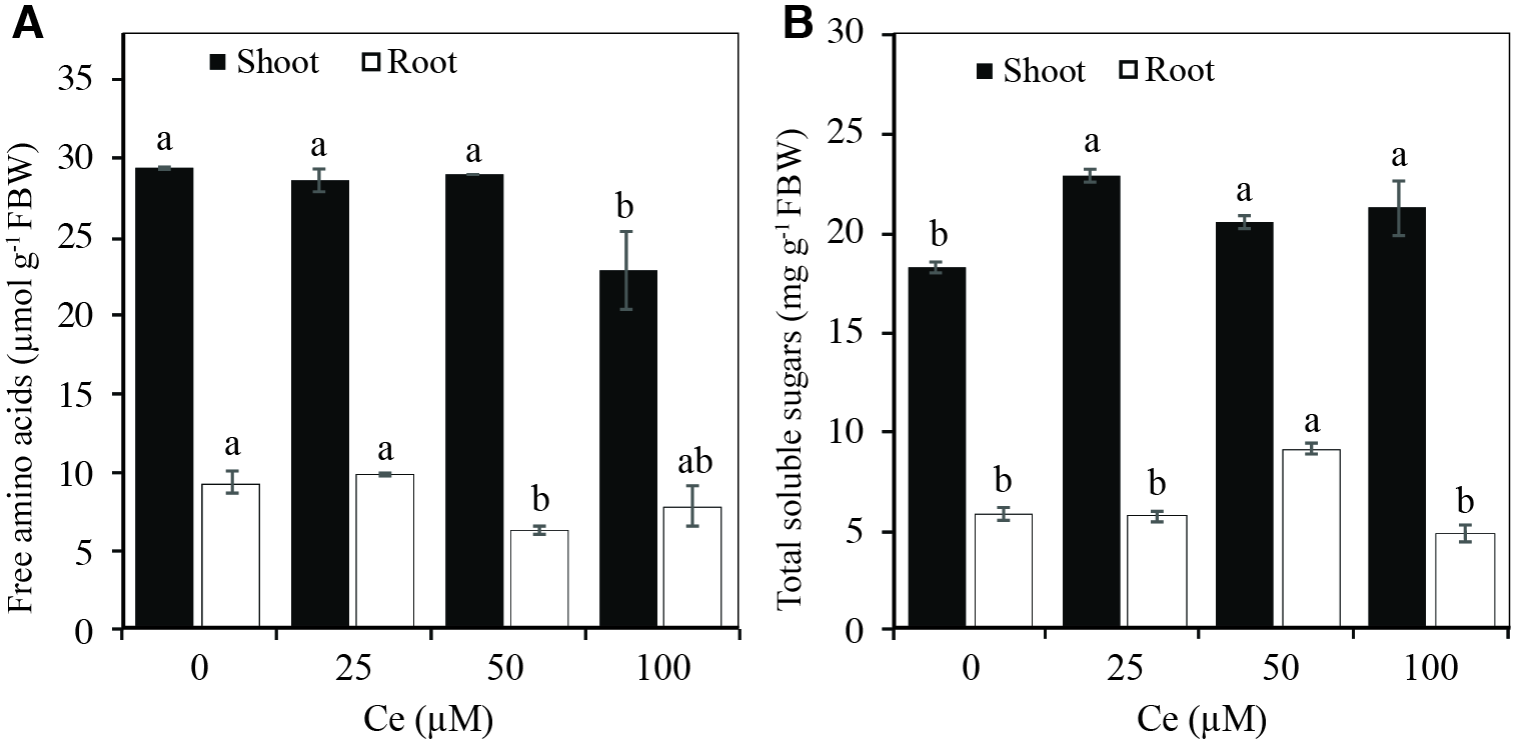
Concentrations of amino acids and sugars in rice plants in response to Ce. Concentration of total free amino acids (A) and total soluble sugars (B) in shoots and roots of rice cv. Morelos A-98 plants treated with 0, 25, 50, or 100 μM Ce. Cerium was supplied as CeCl_3_ 7H_2_O. Bars represent means of 26-d-old plants grown in the nutrient solution for 28 d. Means ± SD with different letters in each variable indicate statistical differences among treatments (Duncan, *P* ≤ 0.05).

The concentration of total free amino acids in shoots decreased by 23.2% with the application of 100 μM Ce. In roots, the application of 50 μM Ce decreased the concentration of amino acids by 32.3% with respect to the control (Fig 7A). The concentration of total sugars in the shoots increased by 25, 12, and 16% with the application of 25, 50, and 100 μM Ce, respectively, in comparison with the control. In roots, there was a significant increase of 58.3% in the concentration of sugars with the addition of 50 μM Ce (Fig 7B).

### Ce differentially affects nutrient concentration in rice

**Fig 8:**
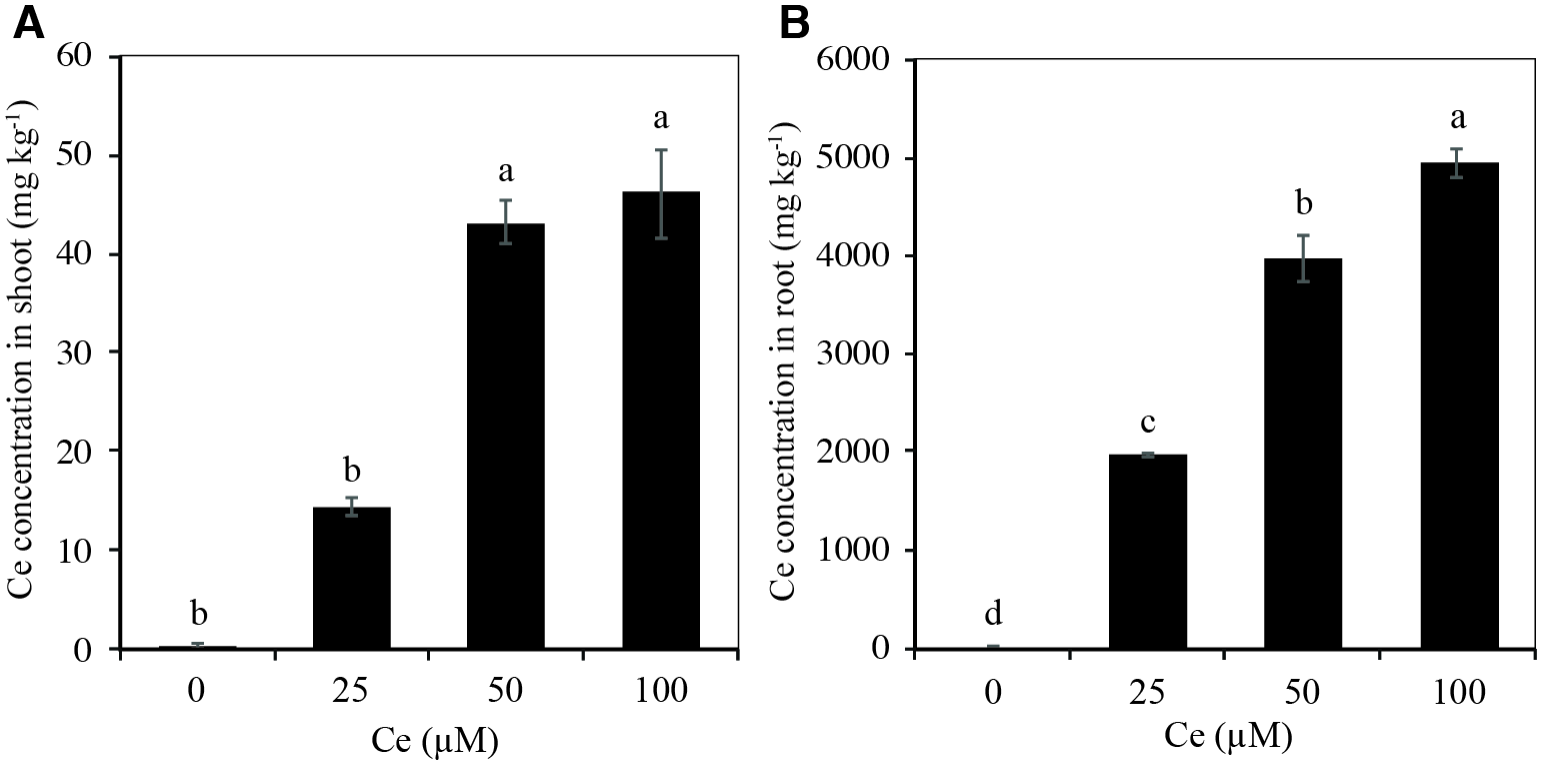
Cerium concentrations in rice plants during the vegetative stage in response to Ce treatments. Ce concentration in shoots (A) and roots (B) of rice cv. Morelos A-98 plants treated with 0, 25, 50, or 100 μM Ce. Cerium was supplied as CeCl_3_ 7H_2_O. Bars represent means of 26-d-old plants grown in the nutrient solution for 28 d. Means ± SD with different letters in each variable indicate statistical differences among treatments (Duncan, *P* ≤ 0.05).

In order to identify a possible interaction between the absorption of Ce and the essential nutrients, the concentrations of Ce, macronutrients, and micronutrients were determined in shoots and roots of rice cv. Morelos A-98 plants grown in different Ce concentrations for 28 d. The Ce concentration in shoots and roots was positively related with the Ce concentration in the nutrient solution. Moreover, the Ce concentration was higher in roots (Fig 8B) than in shoots (Fig 8A).

The concentrations of macronutrients (N, P, K, Ca, and Mg) in rice shoots were not affected by the application of Ce (Table 1). In roots, no significant differences were found in the N and K concentrations among treatments (Table 1). The P and Mg concentrations increased significantly in the roots of plants treated with 50 μM Ce, in comparison with the control. These increases were 26.1% for P and 31.25% for Mg. Conversely, the Ca concentration in roots decreased with the application of Ce.

**Table 1:** Macronutrient concentrations in shoots and roots of rice plants treated with Ce for 28 d.

Ce (μM)	Nutrient concentration (g kg^-1^ DBW)				
	N	P	K	Ca	Mg
	**Shoots**				
0	22.93 ± 4.93 a	6.88 ± 0.39 a	27.25 ± 1.5 a	1.75 ± 0.15 a	4.08 ± 0.24 a
25	27.68 ± 0.62 a	6.60 ± 0.37 a	28.24 ± 1.2 a	1.83 ± 0.05 a	4.03 ± 0.20 a
50	27.23 ± 1.73 a	6.08 ± 0.60 a	25.78 ± 1.0 a	1.65 ± 0.11 a	3.93 ± 0.29 a
100	28.88 ± 1.16 a	5.73 ± 0.25 a	26.38 ± 2.3 a	1.63 ± 0.05 a	4.05 ± 0.22 a
	**Roots**				
0	11.38 ± 0.61 a	2.29 ± 0.24 b	22.92 ± 1.53 a	1.18 ± 0.03 a	1.58 ± 0.07 b
25	11.11 ± 0.58 a	2.40 ± 0.09 ab	24.39 ± 1.00 a	0.91 ± 0.01 bc	1.64 ± 0.03 ab
50	14.11 ± 1.22 a	2.86± 0.16 a	26.15 ± 2.07 a	1.11 ± 0.13 ab	2.14 ± 0.33 a
100	14.18 ± 1.5 a	2.53 ± 0.09 ab	25.35 ± 0.36 a	0.87 ± 0.02 c	1.69 ± 0.02 ab

Cerium was supplied into the nutrient solution as CeCl_3_ 7H_2_O. Means ± SD with different letters in each column per organ indicate statistical differences among treatments (Duncan, *P* ≤ 0.05). DBW: Dry Biomass Weight.

In shoots, the concentrations of the micronutrients Fe, Cu, Mn, and B were not significantly affected by the application of Ce, compared with the control. On the other hand, the applications of 25 and 100 μM Ce increased the Zn concentration by 69.6 and 47.8%, respectively, in comparison with the control (Table 2).

**Table 2:** Micronutrient concentrations in shoots and roots of rice plants treated with Ce for 28 d.

Ce (μM)	Nutrient concentration (mg kg^-1^ DBW)				
	Fe	Cu	Mn	B	Zn
	**Shoots**				
0	136.15 ± 2.16 a	6.90 ± 0.83 a	254.75 ± 38.35 a	22.00 ± 2.03 a	11.53 ± 2.14 b
25	133.33 ± 9.30 a	5.25 ± 0.96 a	257.88 ± 22.07 a	23.75 ± 1.43 a	19.53 ± 0.54 a
50	127.53 ± 3.96 a	6.10 ± 0.97a	254.98± 45.30 a	18.85 ± 1.07 a	12.13 ± 1.06 b
100	127.05 ± 10.36 a	5.38 ± 0.50 a	223.80 ± 12.87 a	20.03 ± 1.34 a	17.03 ± 1.41 a
	**Roots**				
0	810.61 ± 57.09 a	5.13 ± 0.86 a	42.02 ± 3.56 ab	27.81 ± 2.77 a	35.23 ± 4.88 a
25	808.90 ± 11.57 a	7.76 ± 0.51 a	41.65 ± 2.08 ab	40.50 ± 3.57 a	29.84 ± 3.82 ab
50	824.42 ± 76.92 a	8.38 ± 3.55 a	44.72 ± 2.72 a	46.43± 11.33 a	22.07 ± 1.52 b
100	600.40 ± 26.09 b	6.27 ± 0.75 a	34.43 ± 2.10 b	38.59 ± 1.31 a	24.69 ± 0.78 b

Cerium was supplied into the nutrient solution as CeCl_3_ 7H_2_O. Means ± SD with different letters in each column per organ indicate statistical differences among treatments (Duncan, *P* ≤ 0.05). DBW: Dry Biomass Weight.

In roots, none of the Ce concentrations tested had any effect on the Cu, Mn and B concentrations. Conversely, there was an evident negative effect of Ce on the Zn concentration in the roots. The Fe concentration decreased by 25.9% with the application of 100 μM Ce, relative to the control (Table 2).

### Application of Ce results in different correlations with other variables measured

The Ce concentration in shoots and roots was significantly correlated with some variables evaluated in this research. The Ce concentration in shoots (CeS) was positively and significantly correlated with the number of tillers and the dry biomass weight of the shoots. Conversely, it significantly correlated inversely with the chlorophyll *b* and boron concentrations in the shoots. With regard to the Ce concentration in the roots (CeR), positive correlations were observed in the number of tillers, chlorophyll *b* concentration, and N, P, and B concentrations. Moreover, negative correlations with chlorophyll *a*, amino acid concentration in shoots and roots, and Zn concentration in both plant tissues analyzed were observed. There were no significant correlations of CeR with the following variables: plant height, root length, root volume, shoot and root fresh and dry biomass weight, total chlorophyll concentration, total sugars concentration in shoots and roots, and the concentrations of K, Ca, Mg, Fe, Cu, and Mn (Table 3).

**Table 3:** Pearson correlation between the Ce concentration in shoots and roots, and other variables evaluated in rice plants grown in different Ce concentrations for 28 days.

Variables compared	CeS	CeR
PH	-0.36	-0.38
RL	-0.09	-0.14
NT	0.64*	0.53*
RV	0.28	0.16
SWBW	0.42	0.27
RWBW	0.11	0.00
SDBW	0.57*	0.45
RDBW	0.26	0.06
Chl*a*	-0.38	-0.57*
Chl*b*	-0.67*	0.70*
Chltotal	-0.45	-0.61
aaS	-0.40	-0.57*
aaR	-0.47	-0.52
TSS	0.25	0.42
TSR	0.20	0.20
N	0.36	0.54*
P	-0.43	0.51*
K	0.01	0.43
Ca	-0.29	-0.35
Mg	0.06	0.35
Fe	-0.14	-0.33
Cu	-0.11	0.08
Zn	-0.06	-0.63*
Mn	-0.12	-0.24
B	-0.49*	0.49*

Cerium was supplied into the nutrient solution as CeCl_3_ 7H_2_O. Means significantly different at *P* ≤ 0.05. PH: plant height; RL: root length; NT: number of tillers; RV: root volume; SFBW: shoot fresh biomass weight; RFBW: root fresh biomass weight; SDBW: shoot dry biomass weight; RDBW: root dry biomass weight; aaS: amino acids in shoots; aaR: amino acids in roots; TSS: total sugars in shoots; TSR: total sugars in roots; CeS: Ce concentration in shoots; CeR: Ce concentration in roots. Numbers followed by an asterisk (*) indicate statistically significant differences between the compared variables (*P* ≤ 0.05).

## Discussion

The rare earth elements (REE) (Y, Sc, La, Ce, Pr, Nd, Pm, Sm, Eu, Gd, Tb, Dy, Ho, Er, Tm, Yb, and Lu) can have positive effects on seed germination by acting synergically with phytohormones that stimulate germination [4, 35, 1]. In the present research, there were increments in germination ranging from 6.4 to 36.2% with the addition of 4, 8, and 12 μM Ce, with respect to the control (Fig 1). Significant increases in rice seeds treated with Ce in ranges from 2.5 to 20 μg mL^-1^ (7.1 a 61.1 μM Ce) supplied from Ce(NO_3_)_3_ have been reported [7]. However, Barbieri *et al*. [14] found that soaking lettuce (*Lactuca sativa*) seeds in (NH_4_)_2_ Ce(NO_3_)_6_ at concentrations from 5 to 25 mg L^-1^ (9.1 to 45.6 μM Ce) did not affect the germination percentage, although it did improve the initial growth of the seedlings.

The addition of Ce in the initial growth stages showed a marked positive effect on rice plants. There were increases of over 100% in seedling height with all the evaluated concentrations (Fig 2B). These findings are in full agreement with those reported by He and Loh [8], since they found that applying 0.5 μM Ce(NO_3_)_3_ to *Arabidopsis thaliana* plants increased plant height by 60.8%. In our experimental conditions, there were increases in root length and number of over 95% in all evaluated concentrations (Fig 2C and 2D). These results also coincide with the findings reported by Zhang *et al*. [11], who observed that REE participate in the formation of adventitial roots, cell differentiation, and root morphogenesis. Increases in root length from the effect of these elements have also been observed in other species: in *A*. *thaliana* the application of 10 μM Ce(NO_3_)_3_ increased root length by 39.2% [8]; in green gram (*Phaseolus radiatus*) and Chinese cabbage (*Brassica pekinensis*) the addition of Ce(SO_4_)_2_ improved root growth [36].

Shoot and root fresh and dry biomass weights of rice plants increased by more than double with the application of Ce, with respect to the control (Fig 3A and 3B). This could be due to a stimulant effect of Ce in the photosynthetic processes, which resulted in higher biomass production [1, 37, 38]. Significant increases of fresh and dry biomass weight of Ce-treated seedlings have been reported in other studies. In rice plants, the addition of Ce(NO_3_)_3_ in doses between 5 and 15 μg mL^-1^(15.2 and 45.6 μM Ce) increased seedling dry weight by more than 50% [7]. In *A*. *thaliana*, treatment with Ce(NO_3_)_3_ in doses ranging from 0.5 to 2.5 μM increased dry biomass weight by up to 71.5% [8]. Spinach (*Spinacia oleracea*) seedlings treated with 10 μg mL^-1^ Ce(NO_3_)_3_ (30.3 μM Ce) since the germination stage increased fresh and dry biomass weight by up to 38% [12]. In maize, the addition of 20 μM CeCl_3_ increased plant fresh weight by 29.6% [13]. In lettuce seedlings, the application of (NH_4_)_2_Ce(NO_3_)_6_ in doses ranging from 5 to 25 mg L^-1^(9.1 and 45.6 μM Ce) increased dry biomass weight [14]. In contrast with the results obtained in this research, other studies have reported that Ce decreases shoot and root fresh and dry biomass weight. In wheat plants, the application of CePO_4_ in doses from 2 to 25 mg L^-1^ (8.5 a 106.3 μM Ce) for 7 d decreased shoot dry biomass weight by up to 11.5%, and when it was applied for 16 d, the reduction reached 21.1%. In roots, the application of Ce for 7 d caused a decrease in dry biomass weight of up to 41.8%, and when applied for 16 d, the reduction was 34.6% [20]. In maize plants, the addition of 0.5 μM Ce(NO_3_)_3_ decreased root fresh weight by 49%, as well as shoot and root dry weight by 32 and 33%, respectively, while the concentrations in the whole shoots of N, P, S, Ca, Mg, Cl, Cu, Mn, Fe and B in all Ce treatments were generally within the adequate ranges suggested for healthy field-grown corn at a similar growth stage [9]. It has been well documented that plants species may display differential responses to REE according to their genetic background, even considering very close related genotypes. For instance, while Ce stimulated chlorophyll biosynthesis, relative yield and nitrate reductase activity in cowpea plants (*Vigna unguiculata*) [39], it decreased the growth, root function and consequently the nutritional status of mungbean (*Vigna radiata*) [9]. Divergent selection [40], variations in the copy number of REE-responsive genes amongst genotypes [41], and regulatory changes [42, 43] are among the genetic factors determining the differential responses of plants exposed to environmental stimuli such as REE. Nevertheless, the mechanisms behind those responses among plant species, or among varieties of the same species remain poorly understood.

In our experimental conditions, besides evaluating the effect of Ce on seed germination and initial seedling growth, its effect during the vegetative growth stage was also assessed. Ce was found to have significant positive effects on plant height (Fig 4A) and tiller development (Fig 4B). In roots, the low Ce concentrations (25 and 50 μM) did not affect length, while the addition of 100 μM Ce significantly decreased root length (Fig 4C). This same tendency has been observed in other studies. For example, the application of Ce(NO_3_)_3_ in doses from 1 to 15 mg L^-1^ (3.1 to 46 μM Ce) to yellow ginger (*Dioscorea zingiberensis*) stimulated root growth, while the application of 30 mg L^-1^(91.7 μM) decreased growth [11], and the application of 25 and 50 μM Ce increased root volume. In wheat, Hu *et al*. [2] also observed that the application of rare earth elements increased root volume by 12.1%. Shoot fresh and dry biomass weight increased significantly after the addition of 25, 50, and 100 μM Ce without affecting the fresh and dry biomass weight of the roots (Fig 5A and 5B). When increasing concentrations of Ce(NO_3_)_3_ from 50 to 300 mg L^-1^ (153.3 to 919.9 μM Ce), significant increases in dry matter weight both in shoots and roots of pak choi (*Brassica rapa*) plants were observed, while concentrations over 600 mg L^-1^ (1839.8 μM Ce) diminished the values of these variables [18].

Chlorophyll concentrations in leaves were also affected by the application of Ce. Indeed, the addition of 100 μM Ce decreased the chlorophyll *a*, *b* and total concentrations by over 60% (Fig 6). It has been reported that low Ce doses increase the concentrations of photosynthetic pigments in the chloroplasts, and the maintenance of their structure [16, 17]. Similar effects were reported by Chen *et al*. [17], who found that adding 10 to 100 μM Ce(NO_3_)_3_ to suspended cells of ginkgo (*Ginkgo biloba*) gradually increased the concentrations of chlorophylls *a* and *b*, while applying from 500 to 5000 μM Ce(NO_3_)_3_ significantly decreased the concentrations of these molecules. It has been hypothesized that REE may play an indirect role in chlorophyll formation because some of them may function as catalysts or because Ce3+ could enter into the chloroplast and bind easily to chlorophyll, replacing Mg2+ and form Ce-chlorophyll [39, 44]. In spinach plants grown in Mg2+-deficient Hoagland solution, the addition of CeCl_3_ increased the synthesis of organic nitrogen, protein and chlorophyll, while plants treated with 15 μM Ce3+ showed an increase of 84.31% in the chlorophyll content over the control [45]. Higher concentrations of Ce (i.e. 89.206 and 446.030 μM) resulted in a decrease in the chlorophyll contents over the control. Conversely, it has been also observed that Ce can reduce chlorophyll content in *Hydrilla verticillata* [46], which was attributed to a possible formation of chlorophyllase (the enzyme responsible for chlorophyll degradation) [47], damage to the photosynthetic apparatus and disturbance of chlorophyll biosynthesis or its degradation caused by lipid peroxidation [48].

According to Shan *et al*. [49], histidine promoted the accumulation of La, Ce, Pr, and Nd in fern (*Dicranopteris dichotoma*) leaves, and this amino acid has been found to be involved in the capture and translocation of rare earth elements from the roots to the leaves. However, in the present research the concentration of total free amino acids in shoots and roots was not affected by the application of 25 μM Ce, while there were decreases of up to 37% in the amino acid concentration in shoots with 100 μM Ce and roots with 50 μM Ce (Fig 7A). The effect of Ce on the amino acid concentration has also been reported in other research works. For example, adding *n*CeO_2_ at 125, 250, and 500 mg kg^-1^ (726.3, 1452.6, and 2905.1 μM Ce) to wheat plants did not increase the total amino acid concentration in grains [24]. On the other hand, applying *n*CeO_2_ in concentrations of 500 and 1000 mg kg^-1^ (2905.1 and 5810.2 μM Ce) to barley plants significantly increased the total amino acid concentration by 17.08 and 20.45%, respectively. This could indicate the involvement of *n*CeO_2_ in the synthesis of amino acids [25]. It still remains to be elucidated however, the mechanism by which Ce modifies amino acid biosynthesis and accumulation in plant tissues.

We also observed an increase in the concentration of total sugars in shoots with the three Ce concentrations evaluated, while in roots, it was the application of 50 μM Ce that increased the concentration of total sugars (Fig 7B). There are also reports that foliar application of Ce(NO_3_)_3_ in doses of 50, 150, and 300 mg L^-1^ (153.3, 459.9, and 919.9 μM Ce) to pak choi plants increased the concentration of soluble sugars in leaves [18]. Also, in sweet tea trees (*Cyclocarya paliurus*), applying 0.20 mmol L^-1^ Ce(NO_3_)_3_ (200 μM Ce) significantly increased (14.7%) the concentration of soluble sugars in leaves in comparison to the control [50]. Further research in this area should be strengthened for the effects of Ce on the primary metabolism and especially on carbohydrate biosynthesis and degradation in plants.

The absorption of an adequate amount of essential nutrients, especially in the first growth stages, is a determining factor for good crop development and production. In this work, the effect of Ce on the nutrimental status of rice plants during their vegetative stage was evaluated. REE may affect the nutritional status of plants and further regulate their growth. Various studies have reported synergisms and antagonisms between REE like Ce with essential elements [1, 4, 9]. REE may also significantly affect membrane stability, thus influencing the ionic interactions of cells [2, 19]. Ce influences the nutrimental status of shoots and roots of several species [5, 9, 19]. In this research, significant effects of Ce were observed in the concentrations of macro and micronutrients, both positive and negative, especially in roots. In the shoots, there were no changes in the concentrations of macronutrients, although the Zn concentration increased significantly after the application of 25 and 100 μM CeCl_3_ 7H_2_O. In roots, none of the Ce treatments evaluated significantly affected the concentrations of N, K, Cu, Mn, or B, while P and Mg increased significantly with the addition of 50 μM Ce. Moreover, Ca concentration decreased significantly with Ce concentrations of 25 and 50 μM. The Fe concentration decreased with the Ce concentration of 100 μM, while Zn concentration decreased at Ce concentrations of 50 and 100 μM. Similar results were reported in ginkgo [17], which can be attributed to the fact that Ce can interfere with the function of Ca channels, leading to a blockage of this macronutrient since Ce has an ionic radius similar to that of Ca and can compete with and even replace it [6]. Indeed, REE such as Ce affect plant metabolism by regulating the Ca^2+^ level in the cells [51] in a hormetic manner [52]. In wheat, Ce decreased the concentrations of Ca, Mg, Zn, and Mn in shoots, while in roots it decreased the concentrations of K, Ca, Mg, Zn, Cu, and Mn [20]. In shoots of maize plants, adding Ce decreased the concentrations of N, P, Ca, Zn, Mn, Ca, and Zn, while it increased the concentrations of Mg and B; in roots there was no significant effect on the concentrations of N, P, K, Ca, Mg, or Cu, although there was a decrease in the Ca concentration and an increase in the P concentration, which coincides with the data obtained in this work (Table 1). In mungbean shoots, the addition of Ce decreased the concentrations of Cu, Mn, and Fe but increased the concentrations of Mg and Ca [9]. Foliar Ce treatment in Chinese cabbage decreased the concentrations of Cu, Zn, Pb, Cd, and Ni in shoots and leaves [15]. Adding Ce(NO_3_)_3_ to ginkgo decreased the concentrations of Fe and Cu while it increased the Zn concentration [17]. Ce had no effect on the concentrations of macro or micronutrients in the shoots, with the exception of Zn. In the roots, it had synergic effects with P and Mg and antagonistic effects with Ca, Fe, Mn, and Zn. Taken together, there exist species-specific response to Ce, and possibly diverse effects of this element on mineral concentrations in plants.

To evaluate the impact that the Ce concentration has on shoots and roots with regard to the rest of the evaluated variables, a correlation analysis was done. The Ce concentration in shoots was found to correlate positively and significantly with the number of tillers and shoot dry biomass weight, but negatively with chlorophyll *b* concentration and B concentration. In roots, Ce has a positive correlation with the number of tillers, chlorophyll *a* concentration, total free amino acids in shoots and roots, and Zn concentration (Table 3). Rico *et al*. [24] found positive correlations between Ce and growth parameters, and our results agree with the stimulant effect that Ce has on plant growth. Another positive correlation that was found, both in shoots and roots, is between the chlorophyll concentrations (*a*, *b*, and total) and amino acids with root length. There were also positive correlations between nutrients in shoots, especially P with K, Ca, and Mg; in roots, Mn with P, K, Ca, Mg, Fe, and Cu.

It is worth pointing out that REE induce hormesis, a biphasic dose response to an environmental agent characterized by a low dose stimulation or beneficial effect and a high dose inhibitory or toxic effect [53, 54]. Accordingly, beneficial effects of REE are visible only at lower concentrations and not at higher concentrations [52]. While beneficial effects of cerium on plant biology can be attributed to a considerable stimulation of chlorophyll biosynthesis, photosynthetic activity, as well as antioxidative capacity [55], inhibition of plants growth can be attributed to the loss of cellular turgor [56] or to a reduced extensibility of the cell wall [57, 58, 59]. Such inhibitory effects can also be due to a decreasing efficiency of certain enzymes involved in plant metabolism and energy utilization, as well as a decrease in cell division [39, 60].

Summarizing, our results indicate that Ce stimulates plant growth and development, significantly increasing germination and promoting the root development of seedlings. The germination percentage increased by 36.2% on average. Moreover, shoot height and root length and number increased, as did dry and fresh biomass weight of rice seedlings during their initial growth. Also, during vegetative growth, the application of 25 μM Ce increased plant growth, while 50 and 100 μM increased the number of tillers, root volume, and fresh and dry biomass weight of shoots. With 25 and 50 μM, there were no changes in the concentrations of chlorophylls and total free amino acids, but the concentration of total sugars increased; however, at 100 μM Ce, the concentrations of these variables decreased. Ce did not affect the concentrations of macro or micronutrients in shoots despite the increasing levels of this element. In roots, 100 μM Ce decreased the concentrations of Ca, Fe, Mn, and Zn, but increased the Mg concentration. With these results, it is possible to establish that the application of 8 μM Ce is enough to improve germination and initial seedling growth, while 50 μM Ce is adequate for greater plant growth and development during the vegetative stage, without altering the nutrimental status of rice plants. Nevertheless, the effects of Ce on rice grain yield and quality remain to be explored. Furthermore, future studies will be needed to identify the molecular mechanisms responsible for the physiological and biochemical responses observed.
